# Supplementary figures and images for: Relative Risk of Bladder and Kidney Cancer in Lynch Syndrome: Systematic Review and Meta-Analysis
Source: Cancers (Basel). 2023 Jan 13;15(2):506. doi: 10.3390/cancers15020506 (PMC9856836; doi:10.3390/cancers15020506)

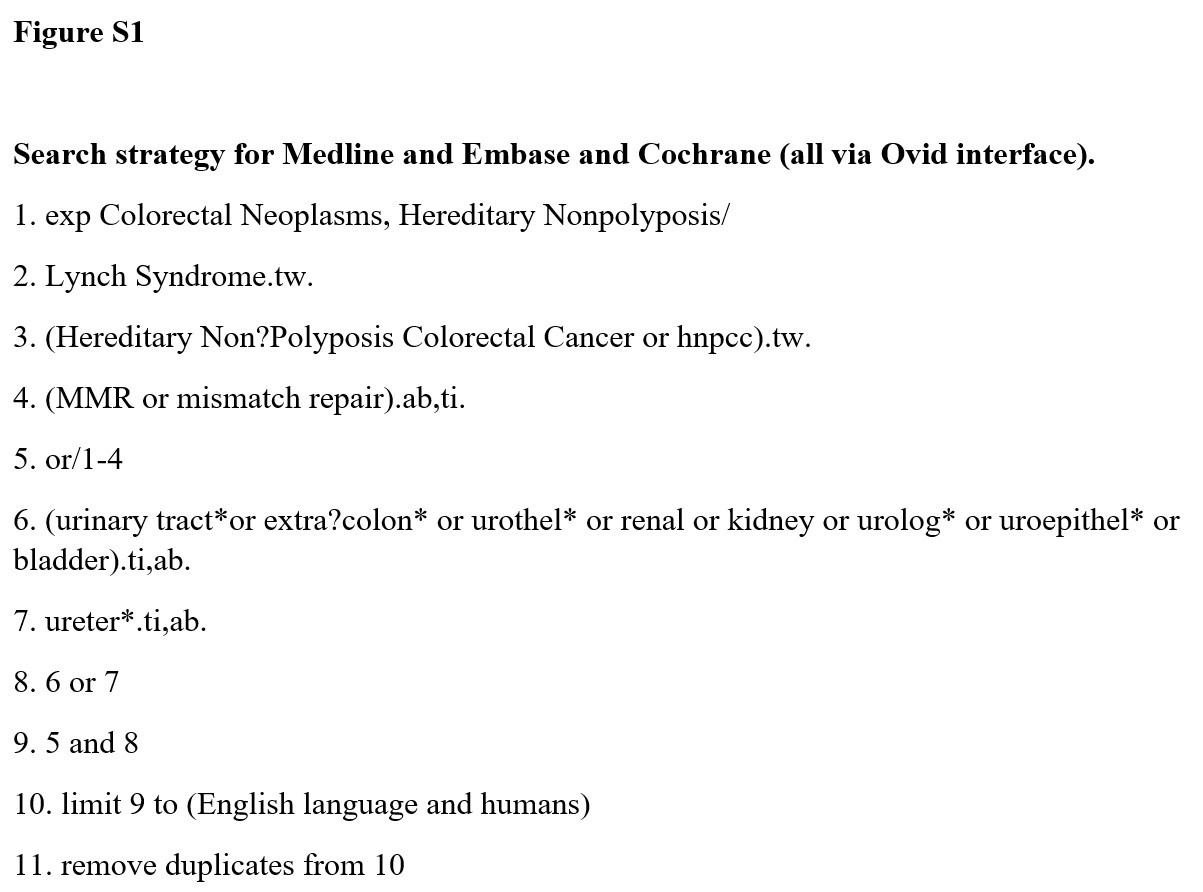

Supplement: Supplementary file 1 [file cancers-15-00506-s001.zip › Figure S1 - Search Strategy.jpg]
